# Multi-criteria decision making to validate performance of RBC-based formulae to screen $\beta$-thalassemia trait in heterogeneous haemoglobinopathies

**DOI:** 10.1186/s12911-023-02388-w

**Published:** 2024-01-02

**Authors:** Atul Kumar Jain, Prashant Sharma, Sarkaft Saleh, Tuphan Kanti Dolai, Subhas Chandra Saha, Rashmi Bagga, Alka Rani Khadwal, Amita Trehan, Izabela Nielsen, Anilava Kaviraj, Reena Das, Subrata Saha

**Affiliations:** 1 Department of Hematology, Postgraduate Institute of Medical Education and Research, Chandigarh, 160012 India; 2 Department of Materials and Production, Aalborg University, 9220 Aalborg, Denmark; 3 Department of Hematology, Nil Ratan Sircar Medical College and Hospital, Kolkata, 700014 West Bengal India; 4 Department of Obstetrics and Gynecology, PGIMER, Chandigarh, India; 5 Department of Clinical Hematology and Medical Oncology, PGIMER, Chandigarh, India; 6 Pediatric Hematology/Oncology Unit, Department of Pediatric Medicine, Advanced Pediatric Centre, PGIMER, Chandigarh, India; 7 Department of Zoology, University of Kalyani, Kalyani, 741235 India; 8 Department of Mathematics, University of Engineering & Management, Action Area III, B/5, Newtown, Kolkata , 700160 India

**Keywords:** $\beta$-Thalassemia carrier screening, Multi-criteria decision making, RBC indices

## Abstract

**Background:**

India has the most significant number of children with thalassemia major worldwide, and about 10,000-15,000 children with the disease are born yearly. Scaling up e-health initiatives in rural areas using a cost-effective digital tool to provide healthcare access for all sections of people remains a challenge for government or semi-governmental institutions and agencies.

**Methods:**

We compared the performance of a recently developed formula SCS$_{BTT}$ and its web application SUSOKA with 42 discrimination formulae presently available in the literature. 6,388 samples were collected from the Postgraduate Institute of Medical Education and Research, Chandigarh, in North-Western India. Performances of the formulae were evaluated by eight different measures: sensitivity, specificity, Youden’s Index, AUC-ROC, accuracy, positive predictive value, negative predictive value, and false omission rate. Three multi-criteria decision-making (MCDM) methods, TOPSIS, COPRAS, and SECA, were implemented to rank formulae by ensuring a trade-off among the eight measures.

**Results:**

MCDM methods revealed that the Shine & Lal and SCS$_{BTT}$ were the best-performing formulae. Further, a modification of the SCS$_{BTT}$ formula was proposed, and validation was conducted with a data set containing 939 samples collected from Nil Ratan Sircar (NRS) Medical College and Hospital, Kolkata, in Eastern India. Our two-step approach emphasized the necessity of a molecular diagnosis for a lower number of the population. SCS$_{BTT}$ along with the condition MCV$\le$ 80 fl was recommended for a higher heterogeneous population set. It was found that SCS$_{BTT}$ can classify all BTT samples with 100% sensitivity when MCV$\le$ 80 fl.

**Conclusions:**

We addressed the issue of how to integrate the higher-ranked formulae in mass screening to ensure higher performance through the MCDM approach. In real-life practice, it is sufficient for a screening algorithm to flag a particular sample as requiring or not requiring further specific confirmatory testing. Implementing discriminate functions in routine screening programs allows early identification; consequently, the cost will decrease, and the turnaround time in everyday workflows will also increase. Our proposed two-step procedure expedites such a process. It is concluded that for mass screening of BTT in a heterogeneous set of data, SCS$_{BTT}$ and its web application SUSOKA can provide 100% sensitivity when MCV$\le$ 80 fl.

**Supplementary Information:**

The online version contains supplementary material available at 10.1186/s12911-023-02388-w.

## Introduction

$\beta$-thalassemia is clinically and epidemiologically one of the most significant hemoglobinopathies prevalent in the Indian sub-continent and results from insufficient ($\beta +$) or no ($\beta _0$) production of $\beta$-globin polypeptide chains caused by a mutation in the $\beta$-globin gene [1–3]. The clinical manifestations of $\beta$-thalassemia are diverse, ranging from asymptomatic microcytic hypochromic red cells in the heterozygous state, known as $\beta$-thalassemia minor or $\beta$-thalassemia trait (BTT) to profound anemia in the homozygous stage ($\beta$-thalassemia-Major or $\beta$-TM), which is fatal in the first few years of life if not supported by regular blood transfusions [4–6]. Occasionally, under conditions of hematopoietic stress, e.g., during pregnancy or intercurrent infections, persons with BTT may also develop anemia and require blood transfusions. But in most instances, these asymptomatic parents are unaware of their carrier status and thus serve as a reservoir of the disease [7–9]. Therefore, carrier screening is inevitable to reduce the burden of the disease [10, 11].

Approximately 5% of the world’s population are carriers of $\beta$-thalassemia genes, particularly in the Mediterranean countries, south-east Europe, Arab nations, Asia, and parts of Africa [12–15]. In India, approximately 10,000 children are born with $\beta$-TM every year, and there are nearly 42 million carriers of BTT, with some communities like Sindhis, Gujaratis, Mahars, Kolis, Saraswats, Lohanas, and Gaurs exhibiting high prevalence [16, 17]. Although the Government of India had included the care and management of patients with thalassemia syndrome, the existing resources and infrastructure remain insufficient [2, 15, 16, 18]. Methods for differential diagnosis between BTT and Iron Deficiency Anemia (IDA) include quantitative detection of Hemoglobin A2 (HbA2) by High-Performance Liquid Chromatography (HPLC) and DNA studies [18, 19]. However, HPLC and DNA tests are expensive, and tests for a large population at risk can make a substantial healthcare burden. Therefore, developing cost-effective screening formulae remains a priority research focus. Over the years, more than forty formulae based on RBC parameters have been developed, as shown in Table 1.

**Table 1:** Forty-two discriminant formulae proposed in the literature

No.	Study	Discriminating formula	Cut-off	Remarks & sample size
1	S & B	$\frac{MCH}{RBC}$	<3.8	For thalassemia minor 9 times out of 10, the cut-off value is below, but not applicable in hemodilution and decreased RBC production(SS: 500)
2	E & F	$MCV-RBC-5 Hb$	<0	Discriminant function identifies 99% of the cases studied but not applicable in pregnancy (SS: 72)
3	Mentzer	$\frac{MCV}{RBC}$	<13	Mentzer classified the highest number of patients correctly (SS: 103)
4	RBC	*RBC*	>5	The measurement of serum iron concentration and iron-binding capacity are needed for the reliable diagnosis of IDA (SS: 122)
5	S & L	$\frac{MCV^2 \times MCH}{100}$	<1530	The false-positives rate was 4.4% (SS:25,302)
6	RDW	*RDW*	<14	Determination of variation of red cell size by erythrography is a rapid and reliable way to distinguish thalassemia minor (SS:85)
7	Ricerca	$\frac{RDW}{RBC}$	<4.4	The sensitivity for the formula is 98% (SS:398)
8	G & K	$\frac{MCV^2 \times RDW}{100Hb}$	<65	Use of red cell volume dispersion results in enhanced accuracy for distinguishing IDA from $\beta$-TM (SS:102)
9	Das Gupta	1.89RBC - 0.33RDW - 3.28	$>0$	Along with the formula and the condition RDW>17.1 recommended for screening (SS:111)
10	MCHD	$\frac{MCH}{MCV}$	<0.34	MDHL provided powerful screening for discriminating
11	MDHL	$\frac{MCH\times RBC}{ MCV}$	>1.75	between IDA and thalassemia (SS: 96)
12	Jayabose	$\frac{MCV\times RDW}{RBC}$	<220	RDW index ensures highest Sens. and Spec. (SS: 102)
13	Huber-Herklotz	$\frac{MCH\times RDW}{10 RBC}+ RDW$	$<20$	Huber-Herklotz can be used to predict TT with high accuracy (SS:114)
14	Sirdah	$MCV-RBC-3 Hb$	<27	Sirdah, G &K or RDWI might be useful in early mass-screening programs (SS: 2196)
15	Kerman- II	$\frac{MCV\times MCH}{ RBC}$	<300	Kerman-I formula presented best outcome
16	Kerman- II	$\frac{MCV\times MCH \times 10}{ RBC\times MCHC}$	$<85$	in screening $\beta$-TM (SS:82)
17	Ehsani	$MCV-10 \times RBC$	<15	Mentzer and Ehsani formulae presents highest YI (SS:284)
18	Keikhaei	$\frac{Hb \times RDW \times 100}{RBC^{2} \times MCHC}$	$>1.27$	Keikhaei, G &K, RDW and E &F formulae demonstrates most reliable discrimination in BTT and IDA (SS:823)
19	Wongprachum	$\frac{MCV\times RDW}{RBC} - 10 Hb$	<104	The formulae can be used as proxy indicators if none sophisticated laboratory are available (SS:234)
20	Nishad	$0.615 MCV+0.518 MCH +0.446 RDW$	<59	Higher Sens is achieved for Ehsani formula, but Spec.is higher for Nishad (SS:326)
21	Sehgal	$\frac{MCV^{2}}{RBC}$	<972	Sehgal and Mentzer formulae showed the best combination in predicting $\beta$TT (SS: 543)
22	Sargolzaie	$$\begin{array}{c} 125.6 + (44.3 \times RBC)\\ -(20.9 \times Hb)-(2.5 \times MCV)\\ +(20.3 \times MCH)\\ -(12.18 \times MCHC) \end{array}$$	$<0.5$	Evaluation of specific information of each region is necessary for discriminating between BTT and IDA (SS:177)
23	Pornprasert	MCHC	<31	Sirdah and Srivastava proved reliable results for discrimination between BTT and IDA (SS: 77)
24	Sirachainan	$1.5Hb-0.05 MCV$	>14	Sirachainan demonstrates best AUC score from identifying IDA and thalassemia traits (SS: 345)
25	Bordbar	$|80-MCV|\times |27-MCH|$	>44.76	Higher Sens is achieved for Bordbar and S &L, and higher Spec. is achieved for Bordbar and Sirdah (SS: 504)
26	Hameed & Hisham	$MCH \times HCT \times \frac{RDW}{(RBC \times Hb)^2}$	<220	Hameed & Hisham was the highest and most reliable in
27		$MCH \times \frac{RDW}{RBC}$	<67	differentiating BTT from IDA (SS: 600)
28	Matos	$1.91 \times RBC + 0.44 \times MCHC$	>23.85	Developed formula provides excellent performance and great diagnostic accuracy (SS: 291)
29	Ravanbakhsh-F1	$\frac{MCV}{HCT}$	<2	Best performing discriminating formulae:
30	Ravanbakhsh-F2	$RDW-3RBC$	$<1.5$	G &K, Keikhaei, RDWI, and E &F are best in terms of YI (SS: 227)
31	Ravanbakhsh-F3	$MCV\times RDW-100RBC$	$<600$	
32	Ravanbakhsh-F4	$\frac{MCV\times Hb}{RDW\times RBC}$	$<10$	
33	Zaghloul-1 & 2	$Hb \times HCT + RBC$	>52.5	E &F and Zaghloul-1 outperforms in discriminating men E &F and RDW outperform for women data set (SS: 249)
34		$Hb \times HCT + RBC - RDW$	>37.1	
35	Kandhro-1 & 2	$\frac{RBC}{HCT} + 0.5 \times RDW$	<8.2	Mentzer, E &F, G &K, RDWI, Ricerca, and Huber are reliable
36		$\frac{5RDW}{RBC}$	$<16.8$	formulae for ease of use in the general population (SS: 610)
37	Merdin-1 & 2	$\frac{RDW \times RBC}{MCV}$	$>1.27$	RDWI, Alparslan and Merdin-1 demonstrated
38		$\frac{RDW \times RBC\times Hb}{MCV}$	$>14.7$	highest YI (SS: 40)
		$$\frac{0.66(MCH-27.0)}{3.9} +0.98$$		
39	Cruise & Index26	$MCHC + 0.603RBC$	$\ge$42.63	Index26 outperforms existing discriminating formulae and can
		$+ 0.523RDW$		be useful to discriminate between IDA and BTT (SS: 907)
40		Combination 26 formulae	$\ge$ 16	
41	Janel (11T)	Combination 11 formulae	$\ge$ 8	11T demonstrates best percentage of correctly identified patients between IDA and BTM (SS: 129)
42	$SCS_{BTT}$	$$\begin{array}{c} 0.2815MCV+ 0.2015MCH\\ - 0.2641RBC- 0.1693RDW\\ + 0.0835Hb \end{array}$$	<24.99	MLP and decision tree algorithm can jointly ensure 100% sensitivity (SS: 1076)

Formulae: S &B: [20]; E &F: [21]; Mentzer: [22]; RBC: [23]; S &L: [24]; RDW: [25]; Ricerca: [26]; G & K: [27]; Das Gupta: [28]; MCHD & MDHL: [29]; Jayabose: [30]; Huber-Herklotz: [31]; Kerman-I & II:[32]; Sirdah: [33]; Ehsani: [34]; Keikhaei: [35]; Wongprachum: [36]; Nishad: [37]; Sehgal: [38]; Sargolzie: [39]; Sirachainan: [40]; Pornprasert [41]; Bordbar: [42]; Hameed & Hisham: [43]; Matos: [44]; Ravanbakhsh-F1, F2, F3 & F4:[45]; Zaghloul-1 & 2: [6]; Kandhro-1 & 2: [46]; Merdin-1 & 2: [5]; Cruise & Index26: [4]; Janel (11T): [47]; $SCS_{BTT}$; [18]; SS: sample size

In recent years, several authors independently evaluated the efficiency of the above formulae [48–51] and reported that most of the formulae suffer from interference with iron and other nutritional deficiency anemia [48, 52]. The sensitivity (SE) and specificity (SP) values of some formulae varied considerably [19, 53–55]. Therefore, it remains critical to understand the strengths and limitations of all the existing discrimination formulae in a heterogeneous data set consisting of different hemoglobinopathies [56, 57]. Performance measures such as SE and SP or PPV and negative predictive value (NPV) represent a trade-off. The SE (or SP) enumerates the ability of a screening formula to identify subjects with (or without) the disease condition correctly. The critical perception is that focusing on only sensitivity when the consequence of a false negative rate is exceptionally high means applying such a formula has an adverse effect. Similarly, if we compare screening formulae directly in terms of area under the curve (AUC), a test with a smaller AUC might also be acceptable in certain circumstances [58]. Consideration of PPV and NPV might remove confusion somewhat, but not all [59, 60]. Consequently, many other performance measures, such as accuracy (ACC) and F1 score, are also recommended. Therefore, a trade-off among possible performance matrices is necessary when recommending the best-performing formula [61].

We aimed to rank forty-two formulae based on eight performance measures: SE, SP, Youden Index (YI), AUC-ROC, PPV, NPV, accuracy, and false omission rate (FOR). Three MCDM methods: (i)Technique for Order of Preference by Similarity to Ideal Solution (TOPSIS), (ii) COmplex PRoportional Assessment (COPRAS), and (iii) Simultaneous Evaluation of Criteria and Alternatives (SECA) were employed. Therefore, the approach we proposed for evaluating formulae can minimize bias. Additionally, as shown in Table 1, some researchers recommend the use of a combination of formulae, e.g., [47] developed *Janel 11T* formula by aggregating the performance of eleven different formulae. The authors suggested a cut-off of 8 out of the 11 existing formulae in favor of BTT (at least 8 formulae recommended BTT). But formulae include some well-performed formulae (e.g., Shine & Lal), thereby questioning the necessity of aggregation and such rigorous evaluation.

The RBC-based discrimination formulae are cost-effective and applicable in low-resource settings but have yet to make the transition to an effective mass-screening decision support system. In this study, first, we evaluated the performance of forty-two formulae on a heterogeneous set of data based on eight different performance measures through MCDM methods. We found that SCS$_{BTT}$ [18] ensured a higher rank in two of the three MCDM methods. With this encouraging result, a modification of SCS$_{BTT}$ was proposed and validated the modification with 939 samples collected from the Nil Ratan Sircar Medical College and Hospital, Kolkata, India. It was found that SCS$_{BTT}$ and its web application, SUSOKA, can be used for mass screening in the Indian context to reduce the cost of a molecular diagnosis for a heterogeneous set of populations.

## Methods

### Population evaluated

This retro-prospective laboratory-based study was conducted in the Hemolytic and Nutritional Anemia Laboratory, Department of Hematology, PGIMER, in collaboration with the Departments of Obstetrics and Gynecology, Clinical Hematology and Medical Oncology and Pediatrics (Pediatric Hematology-Oncology Unit). Active patient recruitment was done between January 2020 to March 2022. Retrospective record mining was done for cases tested between January 2015 to December 2020. The test was conducted on 6,388 samples (5,035 normal subjects NS, 65 HbE, 169 HbD-Punjab/Los Angeles, 203 sickle cell traits (SCT), 194 iron deficiency anemias (IDA) and 722 BTT). Out of the 722 subjects identified as BTT carriers, 40 also had HbE traits (double heterozygous E$\beta$), 17 had concomitant IDA, and 4 were identified as HbDβ. We excluded samples from the following subjects: (i) recently transfused subjects, (ii) subjects in whom the complete hemogram and HPLC data were not available, (iii) subjects who did not have a clear-cut diagnosis, and (iv) subjects with an acute bleeding episode within last three months. Complete blood count (CBC) data were collected during routine diagnostic analysis, and no additional information or extra experiments, such as Vitamin B12 studies, were performed for this study protocol. The laboratory at PGIMER is under the United Kingdom National External Quality Assessment Service (UK NEQAS) Hematology program and BioRad HbA2 EQA program.

### Validation set

The validation set consisted of 939 samples (490 normal individuals, 170 HbE, 4 HbD, and 275 BTT). Out of the last 275 samples, 213 were identified as BTT carriers, 11 had $\beta$-TM, 3 had $\beta$ trait with high fetal hemoglobin, 44 had HbE trait (double heterozygous E$\beta$), and 4 had HbS trait (double heterozygous S$\beta$) collected from the Nil Ratan Sircar (NRS) Medical College and Hospital, Kolkata, India. Active patient recruitment was done between January 2020 to December 2021, and similar exclusion criteria were used.

### Diagnostic performance

Based on the literature, 42 discrimination formulae were considered for evaluation by using the following eight measures: $\text {Sensitivity (SE)}= \frac{TP}{TP+ FN};$ $\text {Specificity (SP)}= \frac{TN}{TN+ FP};$ $\text {Youden's Index (YI)} = TPR+TNR -1$; AUC-ROC = $\frac{1}{2}$-$\frac{FPR}{2}$+$\frac{TPR}{2}$=$\frac{1}{2}- \frac{FP}{2(FP+TN)}+\frac{TP}{2(TP+FN)}$; $\text {Accuracy }(ACC) = \frac{TP + TN}{TP+ TN+ FP+ FN};$ $\text {Positive predictive value (PPV)} = \frac{TP}{TP+ FP};$ $\text {Negative predictive value (NPV)}= \frac{TN}{TN+ FN}$; $\text {False omission rate (FOR)} = \frac{FN}{TN + FN}$ where TP, true positive; TN, true negative; FP, false positive; and FN, false negative. Therefore, *FOR* has a negative impact (i.e., the lower value represents the excellent indicator), and all others have a positive effect (i.e., the higher value means the excellent indicator).

### Statistical analysis

Descriptive statistics, Kruskal Wallis test, Student’s t-test, and ANOVA were conducted where the significance level was set at p<0.01. Statistical analyses were performed using IBM SPSS-27 for Windows (IBM Corp, NY and USA). The SECA method’s optimization problem was solved using Wolfram Mathematica, and the final ranking for all three MCDM methods was done in Python.

### MCDM methods

We present the complete methodology for three MCDM methods, namely, TOPSIS, [62], COPRAS [63, 64], and SECA [65] with a detailed explanation in the Supplementary file (Section B). Notably, the characteristics of the three methods are different [66]. A final rank obtained by the COPRAS method is based on the ratios to the ideal and the anti-ideal solutions, whereas Euclidian distance is considered in the TOPSIS method. The SECA method is characteristically different from the previous two methods as weights for each criterion are determined by solving a non-linear multi-objective optimization problem. We refer to the work by [67], where the authors proposed a framework for formal guidelines for the selection of MCDM methods.

## Results

In this study, we used seven parameters: hemoglobin (Hb), hematocrit (HCT), mean corpuscular volume (MCV), mean corpuscular hemoglobin (MCH), mean corpuscular hemoglobin concentration (MCHC), red blood cell (RBC), and red cell distribution width (RDW) of 6,388 subjects for the performance evaluation of forty-two formulae (Table 1). Samples were divided into multiple groups, and an overview of each parameter in each group is presented in Supplementary file (Section A in Table A1). Applying the Kruskal-Wallis test, we found that all the parameters significantly differed between the groups (p<0.001). The mean values of Hb, MCV, and MCH were higher, and RBC and RDW were lower for the normal subjects (NS) compared to IDA, BTT, SCT, and HbE samples. BTT subjects showed a lower value of Hb, MCV, and MCH and a higher value of RBC and RDW of all the groups. The performance of the formulae on the test data set is presented in Table 2.

**Table 2:** Performance analysis of discriminant formulae

Study	ACC.	SE.	SP.	YI	AUC-ROC	PPV	NPV	FOR
S & B	0.884	0.348	0.977	0.325	0.662	0.719	0.897	0.101
E & F	0.870	0.212	0.983	0.196	0.598	0.685	0.879	0.119
Mentzer	0.898	0.464	0.973	0.437	0.718	0.745	0.913	0.084
RBC	0.871	0.433	0.947	0.380	0.690	0.583	0.907	0.089
S & L	0.783	0.950	0.754	0.705	0.852	0.399	0.989	0.008
RDW	0.660	0.011	0.772	-0.217	0.391	0.008	0.819	0.145
Ricerca	0.374	0.760	0.307	0.067	0.534	0.159	0.881	0.040
G & K	0.869	0.270	0.972	0.241	0.621	0.621	0.886	0.112
Das Gupta	0.695	0.527	0.724	0.251	0.626	0.247	0.899	0.075
Telmissani-MCHD	0.317	0.939	0.210	0.149	0.575	0.170	0.952	0.010
Telmissani-MDHL	0.859	0.164	0.979	0.143	0.571	0.571	0.872	0.126
Jayabose-RDWI	0.876	0.392	0.959	0.350	0.675	0.621	0.902	0.095
Huber-Herklotz	0.837	0.013	0.979	-0.008	0.496	0.097	0.852	0.145
Sirdah	0.889	0.338	0.984	0.322	0.661	0.787	0.896	0.102
Kerman-I	0.892	0.630	0.937	0.568	0.784	0.634	0.936	0.060
Kerman-II	0.899	0.488	0.969	0.457	0.729	0.731	0.917	0.081
Ehsani	0.898	0.475	0.971	0.446	0.723	0.740	0.915	0.083
Keikhaei	0.877	0.311	0.975	0.286	0.643	0.681	0.892	0.106
Wongprachum	0.846	0.286	0.942	0.229	0.614	0.460	0.885	0.109
Nishad	0.892	0.570	0.947	0.517	0.759	0.650	0.928	0.069
Sehgal	0.887	0.497	0.942	0.439	0.720	0.549	0.930	0.067
Sargolzie	0.832	0.269	0.929	0.198	0.599	0.396	0.881	0.112
Pornprasert	0.758	0.387	0.822	0.209	0.605	0.272	0.886	0.095
Sirachainan	0.631	0.111	0.721	-0.169	0.416	0.064	0.825	0.133
Bordbar	0.719	0.832	0.699	0.531	0.766	0.322	0.960	0.028
Hameed	0.150	0.992	0.006	-0.002	0.499	0.147	0.804	0.001
Hisham	0.882	0.351	0.973	0.324	0.662	0.694	0.897	0.100
Matos	0.825	0.302	0.915	0.217	0.609	0.380	0.884	0.107
Ravanbakhsh-F1	0.870	0.449	0.942	0.391	0.696	0.572	0.909	0.087
Ravanbakhsh-F2	0.690	0.330	0.752	0.082	0.541	0.186	0.867	0.103
Ravanbakhsh-F3	0.866	0.390	0.948	0.338	0.669	0.565	0.900	0.095
Ravanbakhsh-F4	0.818	0.891	0.805	0.697	0.848	0.441	0.977	0.018
Zaghloul-1	0.149	0.992	0.004	-0.003	0.498	0.146	0.763	0.001
Zaghloul-2	0.151	0.990	0.006	-0.004	0.498	0.146	0.774	0.002
Kandhro-1	0.372	0.191	0.403	-0.406	0.297	0.052	0.743	0.122
Kandhro-2	0.689	0.360	0.745	0.106	0.553	0.196	0.871	0.099
Merdin-1	0.864	0.585	0.912	0.497	0.748	0.533	0.927	0.067
Merdin-2	0.869	0.366	0.956	0.321	0.661	0.587	0.897	0.098
Cruise	0.363	0.755	0.296	0.051	0.526	0.156	0.876	0.040
Janel (11T)	0.887	0.283	0.991	0.274	0.637	0.840	0.889	0.110
Index26	0.893	0.332	0.989	0.321	0.660	0.839	0.896	0.103
SCS$_{BTT}$	0.726	0.974	0.684	0.658	0.829	0.346	0.993	0.004
**Best**	Kerman-II	Zaghloul-1, Hameed	Janel (11T)	S &L	S &L	Janel (11T)	SCS$_{BTT}$	Zaghloul-1, Hameed
**Worst**	Zaghloul-1	RDW	Zaghloul-1	Kandhro-1	Kandhro-1	RDW	Kandhro-1	RDW, Huber-Herklotz

RDW, Huber-Herklotz, Sirachainan, Hameed, Zaghloul-1, Zaghloul-2, and Kandhro-1 leads to negative YI, i.e., extensively high misclassification costs for those formulae

The results demonstrate that the best-performing formula is Kurman-II in terms of ACC; Zaghloul-1 and Hameed in terms of SE and FOR; Janel 11T in terms of SP and PPV; SCS$_{BTT}$ in terms of NPV; and S &L for YI and AUC-ROC. However, the formula Zaghloul-1 shows the worst performance regarding ACC and SP. RDW indices appear as poor performers in terms of SE and PPV. It is somewhat expected that the index with higher sensitivity might lead to lower SP [68]. However, some indices with higher SP lead to significantly lower specificity. Interestingly, YI for Zaghloul-1 is negative, along with formulae such as RDW, Huber-Herklotz, Sirachainan, Hameed, Zaghloul-2, and Kandhro-1. We removed seven such indices from the final analysis. Note that SP is also an important measure to exclude the samples. If specificity is too low, the samples not having the BTT are often recommended for further evaluation, which contradicts the objective of mass screening to save over-utilization of resources and reduce financial burden. The critical insight is that a single formula fails to ensure all higher performance measures. A trade-off among performance measures is required before recommending any specific formula due to the diversity in performance measures. Consequently, we applied three MCDM methods to obtain the ranking for the formulae as presented in Table 3.

**Table 3:** Final ranking for 35 formulae

Formulae	TOPSIS				COPRAS				SECA	
	$D_i^*$	$D_i^*$	$D_i^*$	Rank	$\frac{{s_{i}}_{min}}{s_{i}}$	$Q_i$	$U_i$	Rank	$W_i$	Rank
S & B	0.088	0.028	0.243	23	0.044	0.407	59.487	16	0.617	16
E & F	0.105	0.013	0.111	34	0.037	0.337	49.306	25	0.553	24
Mentzer	0.072	0.043	0.374	15	0.053	0.467	68.253	9	0.672	8
RBC	0.077	0.038	0.330	17	0.050	0.414	60.422	15	0.618	15
S & L	0.009	0.109	0.927	1	0.528	0.633	92.463	2	0.801	2
Ricerca	0.049	0.076	0.606	6	0.113	0.260	37.934	32	0.394	34
G & K	0.098	0.018	0.156	32	0.040	0.350	51.096	24	0.562	23
Das Gupta	0.068	0.046	0.402	12	0.059	0.315	46.065	27	0.498	29
MCHD	0.034	0.102	0.747	5	0.431	0.362	52.882	23	0.484	31
MDHL	0.112	0.009	0.072	35	0.036	0.292	42.673	30	0.510	26
Jayabose	0.082	0.033	0.285	18	0.047	0.404	59.066	17	0.611	19
Sirdah	0.089	0.028	0.236	24	0.044	0.417	60.906	14	0.627	13
Kerman-I	0.050	0.065	0.567	8	0.075	0.517	75.570	4	0.713	4
Kerman-II	0.069	0.046	0.402	13	0.055	0.475	69.453	7	0.679	6
Ehsani	0.071	0.045	0.387	14	0.054	0.471	68.843	8	0.675	7
Keikhaei	0.093	0.023	0.201	27	0.042	0.382	55.741	22	0.592	21
Wongp.	0.096	0.018	0.159	31	0.041	0.318	46.478	26	0.528	25
Nishad	0.058	0.057	0.495	11	0.065	0.493	72.082	5	0.692	5
Sehgal	0.057	0.056	0.495	10	0.067	0.440	64.240	11	0.643	11
Sargolzie	0.099	0.015	0.135	33	0.040	0.293	42.856	29	0.503	28
Pornprasert	0.085	0.028	0.249	21	0.047	0.288	42.099	31	0.485	30
Bordbar	0.025	0.090	0.781	4	0.159	0.481	70.335	6	0.651	10
Hisham	0.087	0.028	0.245	22	0.045	0.403	58.826	18	0.612	18
Matos	0.095	0.019	0.168	30	0.042	0.301	44.042	28	0.509	27
RF-1	0.075	0.040	0.348	16	0.052	0.418	61.014	13	0.621	14
RF-2	0.095	0.020	0.174	29	0.043	0.218	31.836	35	0.410	33
RF-3	0.083	0.032	0.280	19	0.047	0.390	56.920	20	0.596	20
RF-4	0.014	0.101	0.876	3	0.243	0.585	85.497	3	0.761	3
Kandhro-2	0.091	0.024	0.207	26	0.045	0.232	33.912	34	0.423	32
Merdin-1	0.056	0.058	0.506	9	0.067	0.467	68.200	10	0.663	9
Merdin-2	0.086	0.029	0.254	20	0.045	0.384	56.142	21	0.592	22
Cruise	0.050	0.075	0.599	7	0.111	0.252	36.877	33	0.386	35
Janel(11T)	0.096	0.022	0.184	28	0.041	0.402	58.711	19	0.614	17
Index26	0.089	0.027	0.233	25	0.043	0.425	62.081	12	0.635	12
$SCS_{BTT}$	0.010	0.112	0.922	2	1.000	0.684	100.000	1	0.845	1
**Best**				S & L				$SCS_{BTT}$		$SCS_{BTT}$
**Worst**				MDHL				RF-2		Cruise

**Fig. 1:**
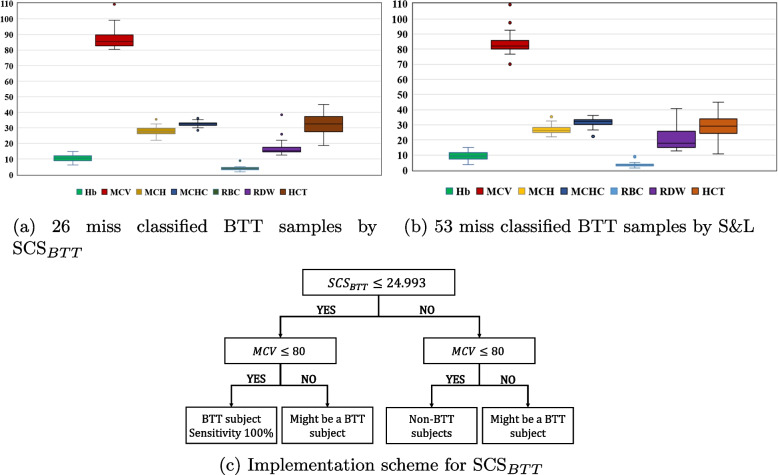
Ranges of seven parameters that lead to false negative measures in discriminating BTTs and implementation scheme

We refer to the Supplementary document for step-wise details on each method. It was found that the S &L formula ensured a higher rank by the TOPSIS method, and SCS$_{BTT}$ ensured a higher rank by the COPRAS and SECA methods. The rationale for such selection is that S &L shows a considerably higher measure of YI, AUC-ROC, NPV, or FOR. Similarly, SCS$_{BTT}$ shows a relatively higher performance measure regarding SE, YI, AUC-ROC, or NPV. We computed Spearman’s rank correlation (see Table B5) among the MCDM methods, which indicated a strong correlation among methods [66] and ensure consistency. Next, we pinpointed the parameter range where the best two formulae miss-classified BTT subjects. We refer to Table B6 for the details of ranges shown in Fig. 1.

Since none of the formulae showed 100% sensitivity (Table 2), we found 26 BTT subjects miss-classified by SCS$_{BTT}$, and among those nine subjects were identified as double heterozygous of HbE & BTT. For S &L, fourteen HbE & BTT subjects appeared amongst the 53 miss-classified samples. However, it appears that SCS$_{BTT}$ can potentially discriminate all BTTs if individual samples have the MCV $\le$80 fl. Note that in Table B6, it is shown that the lower limit of Hb or MCH found for both formulae is relatively low. However, the lower limit for MCV for SCS$_{BTT}$ is above the recommended lower bound in the literature [69, 70]. Therefore, a modification was proposed to the formula SCS$_{BTT}$ to ensure more precise recommendations through a two-step procedure. We hypothesized that SCS$_{BTT}$ can provide 100% sensitivity if $MCV\le 80$ fl. To validate the hypothesis, we used a separate data set, and descriptive statistics for RBC parameters for that validation set are presented in Table A2 in Supplementary file. The results of the validation revealed that only three out of 275 BTT samples were missed by SCS$_{BTT}$ and these three samples had MCV$\ge 80$ fl; one of these samples had double heterozygous $E\beta$. Noticeably, all four that had the HbS trait (double heterozygous $S\beta$) were recommended as BTTs.

## Discussion

The results indicate that a single formula fails to ensure the highest performance with respect to all eight measures. We found five formulae that exhibited the best result for one or two performance measures. This result is consistent with the recent evaluation studies; for instance, [52] reported that the S &L formula ensured the highest SE (we observed the best YI and AUC-ROC for this S &L), the E &F formula presented the highest SP and PPV. The lowest NPV was obtained with the RBC formula. Similarly, [71] reported that the E &F formula showed the highest SE and SP. In this regard, we introduced MCDM methods for ranking the best-performing formula based on eight relevant measures. Since MCDM methods can establish the trade-off among the multiple criteria while determining the final ranking, it helps decision-makers obtain the final Pareto solution. Moreover, the ranking shows the S &L formula is one of the best performers, which is also in line with some evaluation studies [72–74]. The performance of SCS$_{BTT}$ is also consistent with a recent evaluation study from a data set of 2,942 antenatal females samples [72]. Note that while setting weights for TOPSIS and COPRAS, the Shannon-Entropy method is used to assign the related weights to eradicate the bias of the decision marker. The SECA methods are developed so that the weight can be set automatically. The Spearman rank correlation establishes that the final ranking is also almost aligned, demonstrating the utility of the MCDM method’s application in selecting the best formula. Moreover, [75] proposed *clinical utility index* and recommend the use of *Sensitivity* $\times$ *PPV* and *specificity* $\times$ *NPV*, respectively, when positive and negative test results are under scrutiny. Note that the ranking under MCDM methods is also consistent with the newly proposed measure as S &L and SCS$_{BTT}$ both appear within the first quarter.

Notably, discrimination formulae for BTT screening developed based on the principle of binary categorization, and the diagnostic classification of patients depends on whether the measurement of a trait is above or below some specific cut-off point. The rationale behind such variations in recommendation is that individuals with actual levels close to that cut-off point are more likely to be misclassified than intra-individual variability of the underlying traits or due to the influence of uncontrolled covariates. In that sense, the evaluation conducted in this study is exhaustive in terms of the total number of formulae we included and the inclusion of different variants in the test and validation sets. For instance, [48] reported $Index\hspace{3.0pt}26$ is one of the best-performing formulae. However, we find a lower performance measure; this might be due to the variations of samples and the trade-off of multiple measures under consideration. From the perspective of clinical implementation, the validation of $Index\hspace{3.0pt}26$ also needs intense effort. Additionally, the formula might be biased due to the duplication within the twenty-six formulae as reported by [76], Kandhro-II formula [46] is identical to the Ricerca formula [26], and the Keikhaei formula [35] is a duplication of the Jayabose RDW formula [30]. Similarly, $Janel\hspace{3.0pt}11T$ aggregated the performance of eleven indices. Although it outperformed $Index \hspace{3.0pt}26$ in the final ranking, such aggregation introduces complexity in the mass screening process.

Discrimination of BTT in mass screening has been a research priority in recent years. Formulae based on RBC parameters have several advantages, the most important being that they are less expensive for mass screening because RBC parameters are generated automatically during hemogram testing, independent of clinical suspicion or requisition. We found as many as seven CBC parameters used to construct the discrimination formula, as shown in Table 1. In addition, formulae such as $M/H ratio=\frac{\%MicroR}{ \%HypoHe}$ [77] and $MSI=\frac{\%MicroR}{MCV}\times MCHC \times Hb$ [19], and the authors used some parameters that required more costly analyzers. We excluded these formulae from the present evaluation. However, the consensus is that a formula must contain MCV, RDW, and RBC to ensure the best outcomes [76]. According to WHO, if MCV<80 fl, then the samples are to be considered as microcytic anemia^1^. In India, where anemia is an epidemic, the discrimination of BTT is a challenging task. In this context, the recommended range for MCV regarding BTT screening is 50 - 80 fl^2^. Similarly, [78] recommend the range for MCV as < 80 fl for adults; < 70 fl for children six months to six years of age; and < 76 fl in children seven to 12 years of age^3^. [10] recommended that the MCV range should be 60 - 70 fl for BTT carriers. Note that cut-off value for MCV in defining the screening strategy for BTT is widely used; for example, we found the following recommendations for the upper thresholds: $<79$ [79]; $<80$ [69]; $<76$ [80]; and < 76 [70]. Additionally, researchers highlighted the importance of MCV for discriminating BTTs along with other RBC parameters [81–83]. Our study identifies the range as MCV $\le$ 80 fl, also within the recommended bounds. The validation data also support the fact. In the original study [18] to derive SCS$_{BTT}$, the authors emphasize securing 100% sensitivity and considered the infimum and supremum measures for each parameter while defining the cut-off values while implementing the machine learning algorithm. In this study, we undertook extensive validation and found that the formula could apply to heterogeneous samples within thresholds of MCV. Additionally, in the Indian context, individuals with MCV>80 fl but $\beta$-thalassemia carriers are not exceptional [84]. The authors also reported that twenty out of 149 $\beta$-thalassemia carriers in their samples showed HbA2< 3.5%. We also found some samples with similar characteristics (6 samples with HbA2 $\le$4) in our data set. Therefore, excluding samples with HbA2$\le$4 or MCV>80 might mislead the outcome. Accordingly, as we presented in Fig. 1b, we recommend further examination for those individuals to eliminate the risk of spreading.

### Future research

Some limitations of the present study should also be considered. First, because thalassemia data were collected from two hospitals, we did not obtain sufficient data with demographic variables for evaluation. Second, the data available mainly focuses on particular age groups, which could impact the results of our study. Therefore, we need to use sufficient data as proposed by [85] for further evaluation. Further research is warranted to validate its diagnostic value in a population consists of microcytic anemia and various types of anemia.

## Conclusion

Therapies for BTT management not only cost a lot but also need lifelong commitment to sustain life. Early detection through screening based on RBC parameters is thus a feasible cost- and resource-saving option. The results of the present study showed that in a two-step procedure, SCS$_{BTT}$ can classify all the BTT samples with 100% sensitivity when MCV$\le$ 80 fl, even if the sample included borderline cases for HbA2 measure or double heterozygotes. The key conclusions from this study are as follows: a single formula fails to ensure all higher performance measures for screening BTT. Therefore, applying MCDM methods to obtain the final ranking can be an excellent solution to select formulae. Second, formulae-based RBC parameters have several advantages independent of clinical suspicion or requisition. This study observed that an MCV value $\le$ 80 fl can be an essential cut-off for discriminating BTT, but it is insufficient. Therefore, SCS$_{BTT}$ along with the condition MCV$\le$ 80 fl was recommended after conducting validation with data collected from a different institute, and it was found that SCS$_{BTT}$ can classify all the BTT samples with 100% sensitivity when MCV$\le$ 80 fl.

### Supplementary Information

**Additional file 1.**

## Data Availability

All relevant data are available from the authors upon reasonable request to RD and TD.

## Footnotes

1 https://en.wikipedia.org/wiki/Microcytic_anemia

2 https://www.ncbi.nlm.nih.gov/books/NBK531481/

3 https://www.aafp.org/pubs/afp/issues/2009/0815/p339.html
