# Supplementary material for: Multi-criteria decision making to validate performance of RBC-based formulae to screen β-thalassemia trait in heterogeneous haemoglobinopathies
Source: BMC Med Inform Decis Mak. 2024 Jan 2;24:5. doi: 10.1186/s12911-023-02388-w (PMC10759673; doi:10.1186/s12911-023-02388-w)
Supplement: Supplementary file 1 — Additional file 1. [file 12911_2023_2388_MOESM1_ESM.pdf]

Supplementary file for "Multi-Criteria Decision Making to Validate Performance of RBC Based Formulae to Screen β-Thalassemia Trait in Heterogeneous Haemoglobinopathies"

Appendix A Descriptive statistics of RBC parameters

Table A1: Descriptive statistics of RBC parameters in different groups *(Mean $\pm$ standard deviation)* for test data set

Group	Hb	MCV	MCH	MCHC	RBC	RDW	HCT
BTT Female Antenatal (n=117)	9.45 ±1.52	67.41 ±7.69	21.37 ±3.14	31.42 ±1.72	4.41 ±0.89	18.67 ±3.76	29.71 ±4.82
NS Female Antenatal (n=4477)	11.12 ±1.77	86.13 ±9.00	28.48 ±3.63	32.94 ±2.01	3.91 ±0.59	16.44 ±3.67	33.64 ±4.94
HbD Female Antenatal (n=50)	11.18 ±1.95	81.57 ±9.49	27.14 ± 3.87	33.12 ±1.70	4.26 ±0.92	17.56 ±4.55	33.71 ±5.13
SCT Female Antenatal (n=34)	11.28 ±1.79	78.76 ±8.23	26.04 ±3.17	33.00 ±1.19	4.36 ± 0.57	16.51 ±3.29	34.11 ±4.92
HbE Female (n=40)	10.81 ±1.75	73.92 ±7.86	24.18 ±2.99	32.78 ±1.72	4.49 ±0.71	16.70 ±3.70	32.89 ±4.78
HbD Female (n=84)	11.07 ±1.97	80.54 ±11.06	26.49 ±4.62	32.75 ±2.05	4.22 ±0.62	16.97 ±3.50	33.58 ±5.21
SCT Female (n=102)	11.32 ±1.96	79.53 ±11.60	25.89 ±4.10	32.35 ±1.79	4.35 ±0.71	17.35 4.78	34.81 ±5.39
IDA Female (n=191)	7.65 ±1.70	67.79 ±7.94	20.18 ±3.59	29.56 2.46	3.81 ±0.72	21.37 ±4.09	25.73 ±4.78
BTT Female (n=605)	10.14 ±1.51	64.52 ±6.69	20.31 ±2.44	31.29 ±1.68	5.04 ±0.83	18.32 ±3.47	32.41 ±4.70
NS Female (n=529)	11.18 ±2.35	83.59 ±10.98	27.19 ±4.42	32.38 ±2.04	4.13 ±0.76	16.79 ±4.77	34.40 ±6.56
NS Male (n=29)	7.48 ±2.25	61.11 ±6.74	18.19 ±3.01	29.15 ±1.98	4.35 ±1.02	23.09 ±4.05	27.53 ±8.63
IDA Male (n=3)	9.47 ±2.55	54.53 ±3.25	16.70 ±1.85	30.57 ±1.56	5.66 ±1.37	22.37 ±1.60	30.93 ±7.85
SCT Male (n=67)	13.17 ±2.34	81.62 ±10.98	26.68 ±4.29	32.53 ±1.78	4.89 ±0.70	15.70 ±3.67	40.35 ±6.40
HbD Male (n=35)	12.25 ±3.34	81.60 ±15.93	26.93 ±6.23	32.61 ±1.97	4.62 ±1.07	17.38 ±4.19	37.28 ±9.16
HbE Male (n=25)	12.17 ±2.09	74.21 ±6.63	23.83 ±2.49	32.08 ±1.54	5.09 ± 0.66	16.83 ±4.27	37.68 ±6.30

Table A2: Descriptive statistics of RBC parameters in different groups *(Mean ± standard deviation)* in validation data set tested by SCS_{BTT}

	RBC	HB	MCV	MCH	RDW
BTT Female Antenatal (n=15)	4.30 ±0.70	8.93 ±1.16	67.08 ± 6.10	20.72 ±1.72	19.66 ± 3.95
HbE Female Antenatal (n=24)	4.02 ±0.41	10.32 ± 1.03	77.13 ± 5.21	25.89 ±1.83	15.00 ± 1.28
NS Female Antenatal (n=42)	3.86 ±0.49	10.64 ± 1.45	85.56 ± 6.89	27.66 ±2.98	15.33 ±2.80
BTT Female (n=76)	4.97 ±0.75	10.07 ± 1.68	66.17 ± 5.58	20.28 2.45	17.96 ±4.51
HbE Female (n=43)	4.58 ± 0.59	10.89 ± 1.62	73.64 ± 7.40	23.93 ± 3.14	15.43 ± 2.29
NS Female (n=121)	4.12 ±0.59	10.46 ±2.24	80.31 ±8.48	25.41 ±4.31	15.77 ±3.56
BTT Male (n=184)	5.20 ±1.01	10.45 ±2.39	67.02 ±5.38	20.01 ±2.29	18.70 ± 5.14
HbE Male (n=103)	4.95 ±0.67	11.61 ±2.20	73.99 ±6.82	23.59 ±3.10	15.75 ± 2.47
HbD Male (n=4)	4.54 ±0.72	12.13 ±2.53	81.63 ± 5.95	26.68 ±3.07	13.63 ±0.97
NS Male (n=327)	4.54 ±0.79	12.12 ±3.02	83.64 ±9.98	26.59 ±4.54	15.65 ± 3.58

Appendix B The overview of MCDM methods: Entropy weights method

For weight generation for TOPSIS and COPRAS, we used entropy weights method developed by Shannon and Weaver. The method is based on the probability theory and is used to compute uncertain information or entropy. Following are the steps to apply to generates weight:

step 1: We start with the decision matrix, $X = (x_{ij})_{m\times n}$ with $m = 35$ alternatives formula A_1, A_2, $\cdots$, A_m; and $n = 8$ number of criteria (Obtained form Table 2 after exclusion). Consequently, the following decision matrix was constructed:

$$X = (x_{ij})_{m\times n} \tag{B1}$$

Step 2: The entropy value e_j can thus measure the amount of decision information contained in the normalized matrix and queued in each criterion is given by $E_j = -\frac{1}{ln(m)}\sum_{i=0}^{m} p_{i,j} log(p_{i,j})$, where $p_{i,j} = \frac{x_{i,j}}{\sum_{i=0}^{n} x_{i,j}}$ $\forall\{i,j\} \in \{1,..,m\} \times \{1,..,n\}$.

Step 3: Weights (w_j) for each criteria is determined based on the degree of divergence (d_j), and the following relations are used:

(i) $d_j = 1 - E_j$ and (ii) $w_j = \frac{d_j}{\sum_{j=0}^{n} d_j}$ $\forall j \in \{1,..,n\}$.

From Table 2, we obtain the following weights as presented in Table B3, and used for implementing TOPSIS and COPRAS methods.

Table B3: Weights for TOPSIS, COPRAS and SECA

Method	ACC.	SE.	SP.	YI	ROC-AUC	PPV	NPV	FOR
TOPSIS & COPRAS	0.04277	0.19499	0.06984	0.27013	0.01609	0.17586	0.00124	0.22904
SECA	0.10972	0.17126	0.12714	0.13313	0.08239	0.14208	0.07507	0.15919

B.1 TOPSIS

The TOPSIS method is the widely used MCDM methods as it consists of simple computational steps and easy to explain. This method ranks the alternatives according to the distance between the positive and negative ideal solutions. However, an alternative chosen while making a decision is expected to be close to the ideal solution and far from the non-ideal solution. The TOPSIS method consists of the following steps:

Step 1: From the decision matrix ($X = (x_{ij})_{m\times n}$) in Equation (B1), we determine the normalized decision matrix $r = (r_{ij})_{m\times n}$, where $r_{ij} = \frac{x_{ij}}{\sqrt{\sum_{i=1}^{n} x_{ij}^2}}$. Using the weights (w_j), the weighted normalization matrix is calculated as $v = r.diag(w)$, where $diag(w)$ is a diagonal matrix whose the diagonal elements are the weights(w_j) as found Shannon entropy (Table B3).

Step 2: Next, we determine the Ideal (A^+) and Anti-ideal (A^-) solutions using the

following formulas:

$$\begin{aligned}
A^{+} &= \left\{\left(\max_{i}\ v_{ij}|j \in J_1\right), \left(\min_{i}\ v_{ij}|j \in J_2\right)|i = 1, 2, \cdots m\right\} \\
&= \left\{{v_1}^{+}, {v_1}^{+}, \cdots, {v_j}^{+}, \cdots, {v_m}^{+}\right\} \\
A^{-} &= \left\{\left(\min_{i}\ v_{ij}|j \in J_1\right), \left(\max_{i}\ v_{ij}|j \in J_2\right)|i = 1, 2, \cdots m\right\} \\
&= \left\{{v_1}^{+}, {v_1}^{-}, \cdots, {v_j}^{-}, ..., {v_m}^{-}\right\}
\end{aligned}$$

where J_1 and J_2 are the benefit and loss criterion, respectively. Therefore, the ideal solution is considered the best performance, and the anti-ideal solution is considered the worst. Note that in our analysis, seven criteria are benefits criterion, and only false omission rate (FOR) is the loss criterion.

Step 3: The Euclidean distance of each indicator from A^{+} and A^{-} are calculated as follows: $D_i^{+} = \sqrt{\sum_{j=i}^{n}\left(v_{ij} - v_j^{+}\right)^2}$, $\quad D_i^{-} = \sqrt{\sum_{j=i}^{n}\left(v_{ij} - v_j^{-}\right)^2}$, $\forall i = 1, 2, \cdots n$. Using those distance measures, the relative closeness measures ($C_i^{*} \in (0, 1)$) is computed for each alternative, where $C_i^{*} = \frac{S_i^{-}}{S_i^{-} + S_i^{+}}$, $\ i = 1, 2, \cdots m$. By arranging closeness (C_i^{*}) measures, the alternatives are ranked from best to worst (Table 3).

B.2 COPRAS (Complex Proportional Assessment)

[63] was introduced the COPRAS method and was used to evaluate the superiority of one alternative over another and make it possible to compare alternatives. The COPRAS method ranks and evaluates alternatives step-by-step for their importance and utility degree [66]. The steps of the COPRAS method are as follows:

Step 1: From the decision matrix ($X = (x_{ij})_{m \times n}$) in Equation (B1), determine the normalized matrix .

Step 2: Determine the weighted normalised decision matrix $Y = [y_{ij}]_{n \times m}$, where $y_{ij} = w_i x_{ij} \quad i = 1, 2, ..., n; \quad j = 1, 2, ..., m.$

Step 3: Determine the sums of the weighted normalized values for both the beneficial (seven) and non-beneficial (one) criteria. These sums were calculated using Equation (B2) below.

$$K_{+j} = \sum_{i=1}^{n} y_{+ij} \qquad K_{-j} = \sum_{i=1}^{n} y_{-ij} \tag{B2}$$

where y_{+ij} and y_{-ij} are the weighted normalized values of the beneficial and non-beneficial criteria, respectively. Therefore, the higher K_{+j} and lower K_{-j} values represents the better alternatives.

Step 4. The importance of the alternatives is determined by defining the characteristics of the positive alternatives K_{+j} and negative alternatives K_{+j}. Then, the priorities

of the candidate alternatives (C_j) are calculated based on the following formula:

$$C_j = K_{+j} + \frac{K_{-min} \sum_{j=1}^{m} K_{-j}}{K_{-j} \sum_{j=1}^{m} \frac{K_{-min}}{K_{-j}}} \tag{B3}$$

where, K_{-min} is the mean of K_{-j}. Finlay, the C_j values are used for ranking.

B.3 Simultaneous Evaluation of Criteria and Alternatives (SECA)

Unlike other TOPSIS methods, where weights are determined through Entropy calculation, SECA can simultaneously able to determine weight and rank. The final ranking is obtained by executing the following steps:

Step 1: Similar with TOPSIS, we start with the same decision matrix $X = (x_{ij})_{m \times n}$ and all the criterion are divided into two subcategories: beneficial criterion (BC) and non-beneficial (NC). BCs have a positive effect and growth in their values lead to the improvement of the decision- making function, whereas NC have a negative effect and growth in their values have an reverse effect on the objective function. Note that all the criterion used in our model are BC. However, the the normalized decision matrix (X^N) is determined by using the following formula:

$$X^N = \left(x_{ij}^N\right)_{m \times n}, \quad \text{where,} \quad {x_{ij}}^N = \begin{cases} \frac{x_{ij}}{\max_k x_{kj}} & \text{if } j \in \text{BC} \\ \frac{\min_k x_{kj}}{x_{kj}} & \text{if } j \in \text{NC} \end{cases}$$

Step 2: Determine the standard deviation (σ_j, $j = 1, 2, \cdots n$) for each criterion and the correlation between each pair of criteria (π_{jk}) to obtain the variation information within and in-between criterion.

Step 3: Compute the conflict between each criterion against other criteria (π_j), where $\pi_j = \sum_{j=1}^{n}(1 - \pi_{jk})$. Note that, an increase in the variation within the criterion, intensifies the objective importance of that criterion.

Step 4: Normalized the σ and π_j as the reference points by using the following relation.

$$\begin{cases} \sigma_j^N = \frac{\sigma_j}{\sum_{k=1}^{n} \sigma_k} \\ \pi_j^N = \frac{\pi_j}{\sum_{k=1}^{n} \pi_k} \end{cases} \tag{B4}$$

For example, we obtained the following results for MLAs as shown in Table B5.

Step 5: Finally, the weight (w_j) for each criterion is obtained by solving the following

non-linear optimization problem:

$$\begin{cases} \text{Max}Z = \lambda_a - \beta(\lambda_b + \lambda_c) \\ \text{s.t.} \\ \lambda_a \leq S_i, \quad \text{where} \quad S_i = \sum_{j=1}^{n} w_j x_{ij}^N \quad \forall \quad \forall i \in \{1, 2, \cdots m\} \\ \lambda_b = \sum_{j=1}^{n} \left(w_j - \sigma_j^n\right)^2 \qquad \forall i \in \{1, 2, \cdots n\} \\ \lambda_c = \sum_{j=1}^{n} \left(w_j - \pi_j^N\right)^2 \qquad \forall i \in \{1, 2, \cdots n\} \\ \sum_{j=1}^{n} w_j = 1, \qquad \epsilon \geq w_j \leq 1, \forall j \in \{1, 2, \cdots n\} \end{cases} \tag{B5}$$

In Equation (B5), the objective is to maximize the performance of each formulae by considering the impact of the overall performance score of alternative (λ_a), and variation within and between criteria through (λ_b) and (λ_c), respectively. Through the process, the coefficient $\beta \geq 0$ is used for merging objectives, and it the importance of reaching weights for each criterion. Note that the model constraint ensure that the sum of weights should be equal to unit, and $\epsilon = 0.001$ is the lower bound for each criterion. We find the final weight based on the results presented in Table B4.

Table B4: Criteria weights for different β value

β	w_1	w_2	w_3	w_4	w_5	w_6	w_7	w_8
1	0.1199	0.1622	0.1442	0.1728	0.0714	0.1743	0.0233	0.1319
1.5	0.1148	0.1667	0.1357	0.1529	0.0769	0.1582	0.0492	0.1456
2	0.1131	0.1683	0.1328	0.1463	0.0787	0.1528	0.0578	0.1501
3	0.1117	0.1695	0.1305	0.1410	0.0802	0.1485	0.0647	0.1538
5	0.1108	0.1703	0.1290	0.1375	0.0812	0.1456	0.0694	0.1562
8	0.1104	0.1707	0.1283	0.1357	0.0817	0.1442	0.0717	0.1574
10	0.1102	0.1708	0.1280	0.1352	0.0818	0.1438	0.0724	0.1578

From Table B4, we can see that the weights for each criterion become stable when $\beta \geq 5$ [65].

B.4 Additional results

Table B5: Spearman's rank correlation coefficient

	COPRAS	SECA
TOPSIS	0.97675	0.95294
COPRAS	-	0.98067

Table B6: Parameters ranges for BTT samples miss-classified by S&L and SCS$_{BTT}$

Formula	SCS_{BTT}	$S\&L$
Hb	6.2 − 14.9	3.7−14.9
MCV	80.5−109.4	70−109.4
MCH	22−35.7	22−35.7
MCHC	28.5−36.2	22.4−36.2
RBC	2.75−8.87	2.29−8.87
RDW	12.6−38.4	12.6−40.8
HCT	18.6−39.8	10.6−41.3
